# Waterpipe and Co-Use of Inhaled Nicotine and Tobacco Products: Findings from a Population-Based Cross-Sectional Household Survey in Germany

**DOI:** 10.1093/ntr/ntaf192

**Published:** 2025-09-17

**Authors:** Stephanie Klosterhalfen, Neal D Freedman, Daniel Kotz

**Affiliations:** Institute of General Practice (ifam), Addiction Research and Clinical Epidemiology Unit, Centre for Health and Society (chs), Medical Faculty and University Hospital Düsseldorf, Heinrich Heine University, Düsseldorf, Germany; Division of Cancer Control and Population Sciences, National Cancer Institute, Rockville, MD, United States; Institute of General Practice (ifam), Addiction Research and Clinical Epidemiology Unit, Centre for Health and Society (chs), Medical Faculty and University Hospital Düsseldorf, Heinrich Heine University, Düsseldorf, Germany; Department of Behavioural Science and Health, University College London, London, United Kingdom

## Abstract

**Introduction:**

This study aims to provide prevalence data on dual-use (one additional product) and poly-use (two or more products) of inhaled nicotine and tobacco products (cigarettes, e-cigarettes and/or heated tobacco products (HTP)) among current waterpipe (WP) users in Germany over recent years, and explores specific person characteristics associated with such co-use.

**Methods:**

A series of cross-sectional, nationwide, representative, face-to-face household surveys in Germany between 2019 and 2023. Samples were selected using multistratified random sampling (50%) combined with quota sampling (50%). The current analysis included 61 713 respondents (aged ≥14 years) of which 1303 reported current WP use.

**Results:**

WP use in Germany fluctuated between 1.5% and 2.8% from 2019 and 2023. The prevalence rate for 2023 was 1.5% (95%CI = 1.2,1.7). Among users, exclusive WP use declined from 41.8% (95%CI = 35.3,48.5) in 2019 to 26.3% (95%CI = 20.2,33.3) in 2023. Over this period, dual-use increased from 49.3% (95%CI = 42.6,56.1) to 62.3% (95%CI = 55.0,69.3), and poly-use rose from 8.9% (95%CI = 5.5,13.4) to 11.2% (95%CI = 7.1,16.7). Multivariable analyses confirmed a significant decline in exclusive WP use over time (OR per year = 0.90, 95%CI = 0.81–0.99), while the increases in dual-use was not statistically robust after adjustment. Among dual users, the majority (94.2%) reported also smoking cigarettes. The likelihood of using at least one additional product alongside WP was positively associated with increasing age, and with low and middle compared with high educational attainment.

**Conclusion:**

Among current WP users in Germany, exclusive WP use has decreased in recent years, with potential commensurate increase in dual- and poly-use.

**Implication:**

These novel data help inform on the consumption behavior of current WP users and highlights the need for targeted public health interventions that address not only exclusive product use but also the rising combination of different products.

## Introduction

There is evidence of the growing use of waterpipe (WP) globally, particularly among adolescents and young adults.^1-4^ Alongside WP, other nicotine and/or tobacco containing products, such as e-cigarettes and heated tobacco products (HTPs), have gained popularity, often used concurrently with cigarettes.^5-9^ Concurrent use, defined as the use of at least two nicotine or tobacco products within a given timeframe,^10^ is common and may increase harm compared with exclusive use.^5^^,^^6^^,^^11-13^ Combining WP with other inhaled nicotine products may further increase nicotine dependence, reduce cessation intentions, and prolong tobacco use.^14-18^ WP smoking exposes users to harmful toxicants at level similar to cigarette smoking.^19^^,^^20^ A systematic review reported that dual-use of e-cigarettes and cigarettes is at least as harmful as exclusive cigarette smoking, although the certainty of evidence was judged to be low.^14^

In Germany, approximately 30% of adults currently smoke tobacco, predominantly cigarettes.^21^^,^^22^ WP use remains a particular concern among youth, with current (30-day) use prevalence of approximately 4% among 12-17-year-olds, and around 11% among 18-25-year-olds, compared with 2-4% in the general population.^22-24^ E-cigarette use follows a similar pattern, with prevalence between 4% and 7% among 12-17-year-olds, and between 8% and 12% among 18-25-year-olds, while HTP use remains less common (12-17-year-olds = 0.3%, 18-25-year-olds = 3.4%).^24^ Although data from 2021 indicate that around one-third of WP users in Germany also use conventional tobacco products like cigarettes,^25^ little is known about broader patterns of co-use involving e-cigarettes and HTPs. Most existing studies have focused solely on WP-cigarette co-use, leaving a gap in understanding the prevalence and correlates of exclusive, dual-, and poly-use in this population.

WP users are particularly interesting group to examine because WP consumption is comparatively inconvenient, potentially making the addition to of more portable nicotine/tobacco products more attractive. Such combinations could increase the risk of sustained nicotine dependence in WP users more than in users of other products. WP use may also be affected by the COVID-19 pandemic, particularly during lockdown periods when WP cafés were closed, making it important to understand how WP pattern shifted in recent years.

This study addresses this gap by analyzing nationally representative data from Germany to assess the prevalence of current use of cigarettes, e-cigarettes, and HTPs among current WP users. We distinguish between exclusive, dual-, and poly-use patterns and examine sociodemographic and behavioral characteristics associated with each. In addition, we explore changes in these prevalence patterns across survey years at the population level. Understanding these patterns is essential to inform targeted public health interventions and tobacco control strategies tailored to WP users.

## Methods

### Survey Data

Cross-sectional survey data were collected from a representative national household survey which collects data on the use of tobacco and nicotine products in the German population (debra-study^26^;). Data are collected by a specialist market research institute every other month through computer-assisted face-to-face household interviews in a sample of approximately 2000 people aged 14 or older, resulting in six waves of data collection starting in January and ending in November each year. Respondents are selected by random stratified sampling (50%) and quota sampling (50%); details: https://osf.io/s2wxc. The study was approved by the medical ethics committee of the Heinrich-Heine-University Düsseldorf (HHU 5386R). For the current analysis we included data from waves 16 to 45 (January 2019 to November 2023). A detailed analysis plan had been registered prior to analyzing the data: https://osf.io/scze2. A total of 61 713 people were interviewed during this period.

## Measurements

The general DEBRA survey with all questions and response options is openly accessible (https://osf.io/snm3p.)

### Use of inhaled nicotine and tobacco products

#### WP Use

Current WP use was defined as reporting current use of WP. Participants were classified as current WP users if they respond *“Yes, I still use them today”* to the question: “Have you ever used a waterpipe?” (response options 1 = Yes, I still use them today, 2 = Yes, I used to use them regularly, but not anymore, 3 = Yes, I used to use them, but I don’t use them anymore, 4 = No, I have never used them, 5 = No response).

Additionally, we assessed the frequency of WP use. These responses were coded as “less than once per week” or “at least once a week”.

#### Cigarette Use

Current cigarette use was defined as smoking cigarettes daily or less than daily. Participants were classified as current cigarette smokers if they selected either of the first two options to the question: *“Which of the following applies to you best? Please note that smoking means smoking tobacco and not electronic cigarettes or heated tobacco products.”* (1 = I smoke cigarettes every day, 2 = I smoke cigarettes but not every day, 3 = I do not smoke cigarettes at all, but I do smoke tobacco of some kind (e.g., pipe or cigar), 4 = I have stopped smoking completely in the last year, 5 = I stopped smoking completely more than a year ago, 6 = I have never been a smoker (i.e., smoked for a year or more), 7 = No response). Analyses were restricted to cigarette users, as cigarettes are far more common than other smoked products in Germany.

#### E-Cigarette Use

Participants were classified as current e-cigarette user if they respond *“Yes, I have used them until today”* to the question *“Have you ever used an e-cigarette or a similar product (e.g., ELFBAR, Blu, e-shisha, or e-cigar)?”.* (1 = Yes, I have used them until today, 2 = Yes, I used them regularly in the past, but don’t use them anymore, 3 = Yes, I tried them earlier, but don’t use them anymore, or 4 = No, I have never used them, 5 = No response).

#### Heated Tobacco Product (HTP) Use

Participants were classified as current HTP users if they respond *“Yes, I have used them until today”* to the question:*“Have you ever used such a heated tobacco product (IQOS, Glo or Ploom Tech, or a similar product)?”*(1 = Yes, I have used them until today, 2 = Yes, I used them regularly in the past, but don’t use them anymore, 3 = Yes, I tried them earlier, but don’t use them anymore, 4 = No, I have never used them, 5 = No response).

### Concurrent nicotine and tobacco use in current WP users

- *Exclusive use*: Current WP use only (no current cigarette, e-cigarette or HTP use).
- *Dual-use:* Current WP use plus current use of exactly one other nicotine or tobacco product (cigarette, e-cigarette, or HTP).
- *Poly-use*: Current WP use plus current use of at least two other nicotine or tobacco products.

#### Demographics

Demographic variables include: age; sex (female, male); migration background (yes = at least one parent born abroad); region of living (rural <20 000 residents, urban 20 000-500 000 residents, metropolitan >500 000 residents); educational attainment (low = no qualification / junior high school equivalent, middle = secondary school equivalent, high = advanced technical college equivalent / high school equivalent); and monthly net household income per person (low ~ <20^th^ percentile, middle ~20^th^ to 80^th^ percentiles, high ~ >80^th^ percentile).

## Statistical Analyses

Prior to all analyses, this protocol including the analysis plan was pre-registered on the Open Science Framework: https://osf.io/scze2.

To address the proportion of current WP users who concurrently use further nicotine and tobacco products, we reported descriptive prevalence data for each year (aggregated over six waves per year for 2019, 2020, 2021, 2022, and 2023). This includes percentages of exclusive WP use, dual-use, and poly-use, along with 95% confidence intervals (CIs) for the sample of current WP users. Following peer reviewers’ request, we conducted additional logistic regression analyses with survey year as a continuous predictor to assess trends in exclusive WP use, dual-use, and poly-use. Odds ratios (ORs) with 95% CIs were calculated. To explore potential period effects, we ran three models per outcome: an unadjusted model, a core multivariable model (adjusted for age, sex, migration background, income [OECD-adjusted]) and an extended multivariable model (core covariates + education, region, WP use frequency/week). This two-step approach balanced adjustment needs with limited sample size: the core model minimized overfitting, the extended model tested robustness.

To identify the most common combinations of concurrent use, we reported descriptive prevalence data aggregated for waves 16 to 45 (January 2019 to November 2023) on the eight combinations of current WP use with cigarettes (yes/no), e-cigarettes (yes/no), and HTPs (yes/no). Percentages along with 95% CIs were calculated for the sample of current WP user.

To estimate the total effect of predefined respondent characteristic—age (continuous), region of living (rural, urban, metropolitan), migration background (yes / no) frequency of use (less than once per week / at least once per week), educational attainment (low / middle / high), sex (male / female), and income (low / middle / high)—on the likelihood of being a co-user of at least one additional nicotine or tobacco product (yes / no), we employed directed acyclic graphs (DAGs) to identify an appropriate set of adjustment variables. We used the online software DAGitty (https://dagitty.net/) to determine a minimally sufficient adjustment set, ensuring that all non-causal (i.e., biasing) paths between exposure and outcome where blocked while preserving the total effect. The final adjustment set was selected to control for confounders while avoiding adjustment for potential mediators or collider variables. The adjustment sets for regression analyses were determined according to the DAG-based approach, with further details – including the graphs—published alongside the analysis protocol. Depending on the required adjustment set required for each model, we applied either univariate or multivariable regression models.

All analysis were conducted using unweighted data.

## Results

Of the 61 713 participants interviewed between 2019 and 2023, 225 people stated to be current WP user in 2019, 336 in 2020, 261 in 2021, 295 in 2022 and 186 in 2023, resulting in a total number of 1303 (2.1%) current WP users. Missing data (0.4%, *n* = 218) is not included in this total. Of these 1303 current WP users, 34.8% (*n* = 454) only used WP (exclusive user), 55.5% (*n* = 723) used cigarettes, e-cigarettes or HTPs in concurrent (dual-user), and 9.7% (*n* = 126) used at least two further products in concurrent (poly-user). Characteristic of this final sample are reported in Table 1. The majority of respondents had no migration background (56.9%, *n* = 742), were male (62.8%, *n* = 818), under 40 years of age (85.2%, *n* = 1110), and used a WP less than once per week (69.5%, *n* = 906). Regarding the frequency of WP use, differences were observed between exclusive, dual-, and poly-users. Participants who exclusively used WP reported a higher frequency of use (35.2% at least once per week) compared to poly-user (18.3%).

**Table 1:** Sample Description of Current Waterpipe (WP) Users (Total Sample), Exclusive WP-, Dual-, and Poly-Users

	Total sample^†^ 100% (*n* = 1303)	Exclusive WP users’ 34.8% (*n* = 454)	Dual-users^*^ 55.5% (*n* = 723)	Poly-users^#^ 9.7% (*n* = 126)
**Migration background**				
Yes	32.7 (426)	37.2 (169)	30.6 (221)	28.6 (36)
No	56.9 (742)	51.3 (233)	59.8 (432)	61.1 (77)
**Sex**				
Female	37.2 (485)	39.0 (177)	36.1 (261)	37.3 (47)
Male	62.8 (818)	61.0 (277)	63.9 (462)	62.7 (79)
**Age, year (categories)**				
14-17	6.4 (83)	7.9 (36)	5.0 (36)	8.7 (11)
18-24	38.1 (496)	43.4 (197)	34.9 (252)	37.3 (47)
25-39	40.8 (531)	39.9 (181)	40.9 (296)	42.9 (54)
40+	14.8 (193)	8.8 (40)	19.2 (139)	11.1 (14)
**Educational attainment** ^✜^				
Low	22.9 (298)	14.3 (65)	27.8 (201)	25.4 (32)
Middle	34.4 (448)	31.5 (143)	36.1 (261)	34.9 (44)
High	32.2 (420)	39.0 (177)	28.5 (206)	29.4 (37)
**Net monthly household income (€)** ^’^				
Low	23.0 (300)	20.3 (92)	23.8 (172)	28.6 (36)
Middle	56.8 (740)	57.8 (262)	56.3 (407)	56.3 (71)
High	19.6 (255)	21.8 (99)	18.9 (137)	15.1 (19)
**Region of living** ^~^				
Rural	30.4 (396)	30.0 (136)	30.8 (223)	29.4 (37)
Urban	47.2 (615)	45.4 (206)	47.3 (342)	53.2 (67)
Metropolitan	22.4 (292)	24.7 (112)	21.9 (158)	17.5 (22)
**Cigarette use**				
Ever	74.6 (972)	32.2 (146)	97.0 (701)	99.2 (125)
Never	25.0 (326)	67.0 (304)	2.9 (21)	0.8 (1)
**E-cigarette use**				
Ever	43.7 (570)	24.0 (109)	46.5 (336)	99.2 (125)
Never	56.2 (732)	75.8 (344)	53.5 (387)	0.8 (1)
**Heated tobacco product (HTP) use**				
Ever	14.9 (194)	6.2 (28)	16.6 (120)	36.5 (46)
Never	84.6 (1102)	93.3 (424)	82.8 (599)	62.7 (79)
**Frequency of waterpipe use**				
< Once per week	69.5 (906)	64.8 (294)	70.5 (510)	81.0 (102)
≥ Once per week	29.9 (389)	35.2 (160)	28.5 (206)	18.3 (23)

*Data are presented as percentages (absolute numbers), aggregated for waves 16-45 (01/2019-11/2023).*  ^†^Total sample means all respondents, who stated to be a current WP user. ‘Exclusive WP users means current WP users, who don’t use cigarettes, e-cigarettes or heated tobacco products (HTPs) in concurrent. ^*^Dual-users means current WP users, who use one further nicotine product (cigarettes, e-cigarettes, or HTPs) in concurrent. ^#^Poly-users means current WP users, who use two further nicotine products in concurrent. Data for the total sample are presented as 100% within the column. Differences when calculating the total percentage in column can be explained by missing data on the respective variable.  ^✜^*German equivalents to education attainment listed from lowest to highest: low = no qualification / junior high school equivalent, middle = secondary school equivalent, high = advanced technical college equivalent / high school equivalent.*  ^‘^*Net monthly household income: low = approximately < 20th income percentile, middle = approximately 20th to 80th income percentiles, and high = approximately > 80th income percentile.*  ^~^*Region of living: rural (<20 000 residents), urban (20000-500 000 residents), metropolitan (>500 000 residents).*

### Prevalence of Exclusive WP Use, Dual-Use, and Poly-Use

**Figure 1:**
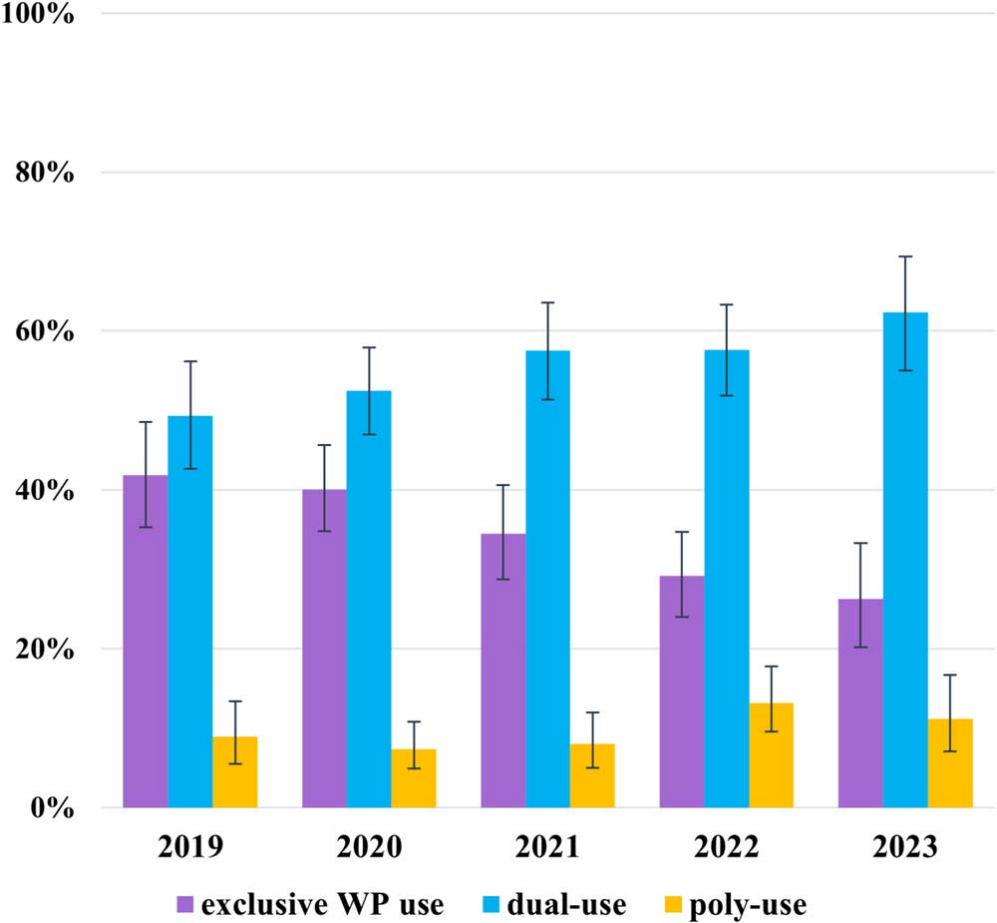
Prevalence of exclusive waterpipe (WP) use, dual-, and poly-use in current WP user between 2019 and 2023. *Bar chart showing the annual prevalence of exclusive waterpipe use, dual-use, and poly-use among current users from 2019 to 2023. Dual-use (waterpipe plus one additional product like cigarettes, e-cigarettes, or heated tobacco) increases over time, while exclusive use declines. Poly-use (waterpipe plus two or more additional products) remains low and stable. Data are based on six survey waves per year, with sample sizes ranging from 186 to 336. Error bars represent 95% confidence intervals*.

Figure 1 shows the prevalence of exclusive WP use, dual-, and poly-use in current WP user between 2019 and 2023. Over time there was a nearly linear decline in exclusive WP use from 41.8% (95%CI = 35.3,48.5) in 2019 to 26.3% (95%CI = 20.2,33.3) in 2023. At the same time dual-, and poly-use increased from 49.3% (95%CI = 42.6,56.1) to 62.3% (95%CI = 55.0,69.3), respectively from 8.9% (95%CI = 5.5,13.4) to 11.2% (95%CI = 7.1,16.7).

Unadjusted analyses showed a decline in exclusive WP use (OR/year = 0.83, 95%CI = 0.76–0.90), and an increase in dual-use (OR = 1.13, 95%CI = 1.04–1.23) and poly-use (OR = 1.15, 95%CI = 1.00–1.32). In the core multivariable models, the negative association between survey year and exclusive WP use remained statistically significant (OR = 0.86, 95%CI = 0.79–0.95), whereas the positive association for dual-use was no longer statistically significant (OR = 1.08, 95%CI = 0.99–1.18). For poly-use, the association with survey year became statistically significant in the core multivariable model (OR = 1.17, 95%CI = 1.00–1.36). In the extended multivariable model including all additional covariates, the downward trend for exclusive WP use remained significant (OR = 0.90, 95%CI = 0.81–0.99), while the trends for dual-use (OR = 1.05, 95%CI = 0.95–1.16) and poly-use (OR = 1.14, 95%CI = 0.97–1.33) were no longer statistically significant. Full results are presented in Table S1.

### Combinations of Current WP Use with Cigarettes, e-Cigarettes and HTPs

Table 2 shows the eight possible combinations of current WP use with cigarettes, e-cigarettes and/or HTPs. The most frequent combination was dual use with cigarettes (*n* = 681/1303, 52.3%, 95%CI = 49.5, 55.0), followed by poly-use with cigarettes and e-cigarettes (*n* = 97/1303, 7.4%, 95%CI = 6.1-9.0). In the subgroup of dual users (*n* = 723), 94.2% (*n* = 681/723, 95%CI = 92.3, 95.8) smoked cigarettes. The co-use with HTPs was uncommon.

**Table 2:** Combinations of Current WP Use with Cigarettes, e-Cigarettes, and Heated Tobacco Products (HTPs)

		Cigarettes yes	Cigarettes no
**E-cigarettes**	**HTP yes**	**1.0%**	**0.0%**
**yes**		(95%CI = 0.5-1.7)	(95%CI = 0.0-0.0)
	**HTP no**	**7.4%**	**2.8%**
		(95%CI = 6.1-9.0)	(95%CI = 2.0-3.9)
**E-cigarettes**	**HTP yes**	**1.2%**	**0.4%**
**no**		(95%CI = 0.6-1.9)	(95%CI = 0.1-0.9)
	**HTP no**	**52.3%**	**34.8%**
		(95%CI = 49.5-55.0)	(95%CI = 32.2-37.5)

*Data are presented as percentages (95% confidence Interval), aggregated for waves 16-45 (01/2019-11/2023). Highlighted in purple = exclusive WP use; highlighted in blue = dual-use (WP + cigarette or e-cigarette or HTP); highlighted in yellow = poly-use (WP + at least two further nicotine products). Differences when calculating the total percentage for total sample can be explained by missing data.*

### Associations of Concurrent Use with Person Characteristics

Table 3 shows the likelihood of the use of minimum one additional nicotine and tobacco product with person characteristics and the frequency of WP use. The likelihood of using at least one additional product alongside WP was positively associated with increasing age (OR = 1.04, 95%CI = 1.02,1.05), and a lower compared with high educational attainment (low: OR = 2.32, 95%CI = 1.65,3.27; middle: OR = 1.49, 95%CI = 1.13,1.97). The likelihood was negatively associated with having a migration background (OR = 0.70, 95%CI = 0.54,0.89), increasing net household income per person (OR = 0.82, 95%CI = 0.71,0.96), and using WP at least once per week compared to people using WP less than once per week (OR = 0.69, 95%CI = 0.54,0.88). To assess whether associations vary depending on the specific tobacco products used, we conducted an additional analysis comparing dual user of WP and cigarette to exclusive WP users. The associations were largely similar (see Table S2).

**Table 3:** Results of Regression Models on Associations Between Respondents’ Characteristics and Concurrent vs. Exclusive WP Use

	Concurrent use^†^ of at least one additional nicotine product alongside WP (yes vs. no)		
	OR	95% CI	*p* value
**Migration background** ^a^			
No	Reference		
Yes	0.70	0.54-0.89	<0.01
**Sex** ^a^			
Female	Reference		
Male	1.12	0.89-1.42	0.34
**Age** (years)*‘*^a^	1.04	1.02-1.05	<0.001
**Educational attainment** ^✜^^b^			
High	Reference		
Middle	1.49	1.13-1.97	<0.01
Low	2.32	1.65-3.27	<0.001
**Region of living** ^~^ ^a^			
Metropolitan	Reference		
Urban	1.24	0.93-1.65	0.15
Rural	1.19	0.87-1.63	0.28
**Net monthly household income** (€)*‘*^c^	0.82	0.71-0.96	<0.05
**Frequency of waterpipe use** ^a^			
< once per week	Reference		
≥ once per week	0.69	0.54-0.88	<0.01

^†^Means, the use of minimum one additional nicotine and tobacco product, *‘*Age and income (OECD equivalent net monthly household income in €) were treated as continuous variables for the regression analyses. ^✜^German equivalents to education attainment listed from lowest to highest: low = no qualification / junior high school equivalent, middle = secondary school equivalent, high = advanced technical college equivalent / high school equivalent. ^~^Region of living: rural (<20 000 residents), urban (20000-500 000 residents), metropolitan (>500 000 residents);

Adjustment sets for regression analyses were derived by application of directed acyclic graphs (DAGs, more details – including the graphs – have been published together with the analysis protocol https://osf.io/scze2):

a Univariate logistic regression model: no adjustment is necessary or possible – as it would produce a collider bias – to estimate the total effect of the independent variable on the outcome.

b Multivariable logistic regression model adjusted for the variable: age.

c Multivariable logistic regression model adjusted for the variables: age, migration background

## Discussion

In Germany between 2019 and 2023, the proportion of co-users of other nicotine and tobacco products among current WP users has increased. In 2023 approximately 74% of current WP users also use cigarettes, e-cigarettes, and/or HTPs. Around half (~52%) of the WP users surveyed also smoke cigarettes. The likelihood of using at least one additional product alongside WP was higher among older people and those with lower educational attainment.

Our findings are align with the trend of concurrent tobacco product use that has been observed for years worldwide.^27-29^ Focusing on current WP users, studies indicate that this group is not homogeneous and exhibit significant differences in their usage behavior regarding exclusive, dual- and poly-use across different regions. For instance, 39% of users in Egypt reported exclusive WP use, compared to 45% in Jordan, and 67% in Palestine in 2016.^30^ In contrast to our data, the group of exclusive WP users had the highest proportion in these countries. We suspect that the high rate of exclusive WP use in certain regions is due to the strong cultural embedding of WP smoking, as well as the overall high prevalence of WP use in these countries, which ranges between approximately 11% and 40%.^31^

When another product is consumed alongside WP in Germany, it is predominantly the cigarette. This is not surprising given the high and stable prevalence and tradition of cigarette smoking in Germany, which has been around 30% for years.^32^ Additional international studies also reveal this correlation of concurrent use of WP and cigarette,^30^^,^^33^ which could be linked to their widespread availability and affordability.

As in other studies, we also observe that people who use at least one additional nicotine and tobacco product in concurrent to WP use (dual- or/ poly-user), use WP less frequently, have lower educational attainment, lower income and are of older age.^33^^,^^34^ However, with respect to age and education, studies have also identified different correlations.^33^^,^^34^ A study conducted in Pakistan found that dual use of WP and cigarettes was associated with younger age and higher educational level.^34^ Lower educational attainment might be linked to the higher prevalence of cigarette smoking among people with less education and lower income in Germany.^35^ A possible explanation may be that people with a migration background may be more likely to use WP more often exclusively and more frequent due to cultural practices. In countries with a cultural tradition of WP use, such as those in Eastern Mediterranean region, dual-use of WP and cigarettes is relatively uncommon. For example, in Lebanon, 35.1% of people currently smoke cigarettes, 38.1% currently use WP, but only 3.6% are dual-user (both WP and cigarettes).^31^

Analyses of our repeated cross-sectional data (2019–2023) indicated a decline in exclusive WP use and increases in dual- and poly-use in unadjusted models. However, only the decline in exclusive use remained statistically significant after adjustment, suggesting that sociodemographic and behavioral factors may partly explain the observed trends. Given the study’s cross-sectional design, these changes reflect population-level patterns rather than individual behavioral shifts. The absence of significance for dual- and poly-use in adjusted models may also be partly due to the small subgroup sizes, which reduce statistical power. The apparent increases in dual- and poly-use should therefore be interpreted with caution and require confirmation in future studies with larger samples or longitudinal designs.

### Strengths and Limitations

Our study has several limitations. First, the limited sample size, due to the relatively low prevalence of WP users in Germany, restricts the ability to perform a more fine-grained, year-by-year analysis of trends concerning all possible combinations of concurrent use of WP with cigarettes, e-cigarettes, and HTPs across different population strata (e.g., gender, specific age groups). As the sample was collected over several calendar years, changes in associations over time cannot be ruled out; however, the small subgroup sizes precluded the inclusion of calendar year and its interaction in all regression models.

Additionally, while grouping concurrent users into a single category simplifies the analysis, we acknowledge that different product combinations may influence consumption patterns and behaviors differently. Due to sample size constraints, we were unable to analyze all dual- and poly-use subgroups separately, which may mask potential differences in their consumption patterns and associated factors. Moreover, we acknowledge that the implications of concurrent use are complex. While dual- and poly-use may increase in harm by reinforcing nicotine dependence and increasing overall consumption, some product combinations—particularly those including non-combustible products —could also serve as a harm reduction strategy. Distinguishing between these patterns requires more detailed data, such as the frequency of use for each product and the sequence of product initiation. Future research should address this important distinction to better understand the health implications of different forms of concurrent use. Due to sample size constraints, we were unable to analyze all dual and poly-use subgroups separately, which may mask potential differences in their consumption patterns and associated factors.

In addition, we excluded current pipe/cigar users from our analyses. While pipe/cigar use is rare in the German population overall, it was somewhat more common among current WP users in our dataset. As these users were excluded, our estimates may slightly underestimate the prevalence of dual- and poly-use and overestimate exclusive WP use.

Social desirability bias may have led to underreporting of WP use, particularly among younger people, due to legal restrictions on using for those under 18. This may have affected the accuracy of self-reported data, potentially underestimating the prevalence of WP use in the population.

Moreover, the absence of data on the duration of WP and other tobacco and nicotine product use, as well as whether WP was the first or primary product used, limits our understanding of the temporal relationship between product initiation and subsequent use patterns. These factors could provide valuable insights into the progression and motivations behind tobacco product use.

The requirement for participants to have sufficient German language skills could result in an underrepresentation of people with a migration background. Furthermore, the detailed information on respondents’ migration backgrounds, preventing the analysis of differences based on place of birth and region of origin, which could have provided insights into potential prior experiences with cultural WP consumption.

The strengths of the study include the use of nationally representative samples and bi-monthly data collection over a five-year period, which provides a substantial dataset for examining changes of concurrent WP use over time. The comprehensive data collection on a wide range of person characteristic and various nicotine and tobacco products, including WPs, cigarettes, e-cigarettes, and HTPs, allows for a more nuanced analysis of concurrent use. Additionally, the use of face-to-face interviews minimize missing data.

## Conclusion

In conclusion, exclusive WP use among current WP users in Germany has decreased in recent years, with potential commensurate increases in dual- and poly-use. These developments highlight the importance of understanding the social and cultural context of WP, cigarettes, e-cigarettes, and HTPs to gain a more comprehensive understanding for targeted public health interventions that address not only exclusive product use but also the rising combination of different products. Further research is needed to determine whether the observed shifts in dual- and poly-use represent robust and stable trends and to explore the interaction between different smoking behaviors, for example focusing on dual-use of WP and cigarettes.

## Supplementary Material

Supplementary_Table_1_ntaf192

Supplementary_Table_2_ntaf192

## Data Availability

The data underlying this article will be shared on reasonable request to the corresponding author.
