# Supplementary material for: Waterpipe and Co-Use of Inhaled Nicotine and Tobacco Products: Findings from a Population-Based Cross-Sectional Household Survey in Germany
Source: Nicotine Tob Res. 2025 Sep 17;28(1):46–53. doi: 10.1093/ntr/ntaf192 (PMC12723216; doi:10.1093/ntr/ntaf192)
Supplement: Supplementary_Table_1_ntaf192 [file supplementary_table_1_ntaf192.docx]

Supplementary Table 1. Results of regression models on associations between respondents’ characteristics and dual use of WP and cigarettes vs. exclusive WP use.

| **Use Pattern** | **Model** | **OR for year** | **95% CI** | ***P* value** |
| --- | --- | --- | --- | --- |
| **Exclusive use´** |  |  |  |  |
|  | Unadjusted | 0.83 | 0.76-0.90 | <0.001 |
|  | Core model^^^ | 0.86 | 0.79-0.95 | <0.05 |
|  | Extended^~^ | 0.90 | 0.81-0.99 | <0.05 |
| **Dual-use^*^** |  |  |  |  |
|  | Unadjusted | 1.13 | 1.04-1.23 | <0.01 |
|  | Core model^^^ | 1.08 | 0.98-1.18 | 0.10 |
|  | Extended^~^ | 1.05 | 0.95-1.16 | 0.32 |
| **Poly-use^#^** |  |  |  |  |
|  | Unadjusted | 1.15 | 1.00-1.32 | 0.05 |
|  | Core model^^^ | 1.17 | 1.00-1.36 | <0.05 |
|  | Extended^~^ | 1.14 | 0.97-1.33 | 0.12 |

*´Exclusive use means current WP use, and no use of cigarettes, e-cigarettes or heated tobacco products (HTPs) in concurrent.
*Dual-use means current WP use, and use of one further nicotine product (cigarettes, e-cigarettes, or HTPs) in concurrent.*

*#Poly-use means current WP use, and use two further nicotine products in concurrent.*

*^Core model: Adjusted for age, sex, migration background, net household income adjusted by OECD equivalence weight.*^~^*Extended model: Core covariates plus educational attainment, region, and WP use frequency per week.*
