# Supplementary material for: Waterpipe and Co-Use of Inhaled Nicotine and Tobacco Products: Findings from a Population-Based Cross-Sectional Household Survey in Germany
Source: Nicotine Tob Res. 2025 Sep 17;28(1):46–53. doi: 10.1093/ntr/ntaf192 (PMC12723216; doi:10.1093/ntr/ntaf192)
Supplement: Supplementary_Table_2_ntaf192 [file supplementary_table_2_ntaf192.docx]

Supplemenary Table 2. Results of regression models on associations between respondents’ characteristics and dual use of WP and cigarettes vs. exclusive WP use.

|  | **Dual use** of WP and cigarettes vs. WP exclusively  (yes vs. no) | | |
| --- | --- | --- | --- |
|  | OR | 95% CI | *P* value |
| **Migration background**^a^ |  |  |  |
| No | Reference |  |  |
| Yes | 0.81 | 0.63-1.02 | 0.07 |
| **Sex**^a^ |  |  |  |
| Female | Reference |  |  |
| Male | 1.16 | 0.92-1.46 | 0.19 |
| **Age** (years) *‘*^a^ | 1.04 | 1.03-1.05 | <0.001 |
| **Educational attainment**^✜b^ |  |  |  |
| High | Reference |  |  |
| Middle | 1.50 | 1.14-1.97 | <0.01 |
| Low | 1.91 | 1.40-2.61 | <0.001 |
| **Region of living**^~a^ |  |  |  |
| Metropolitan | Reference |  |  |
| Urban | 1.15 | 0.87-1.52 | 0.32 |
| Rural | 1.13 | 0.84-1.53 | 0.42 |
| **Net monthly household income** (€)*‘*^c^ | 0.89 | 0.77-1.04 | 0.13 |
| **Frequency of waterpipe use**^a^ |  |  |  |
| < once per week | Reference |  |  |
| ≥ once per week | 0.83 | 0.65-1.05 | 0.12 |

*‘*Age and income (OECD equivalent net monthly household income in €) were treated as continuous variables for the regression analyses. ^✜^German equivalents to education attainment listed from lowest to highest: low=no qualification / junior high school equivalent, middle=secondary school equivalent, high=advanced technical college equivalent / high school equivalent. ^~^Region of living: rural (<20,000 residents), urban (20,000-500,000 residents), metropolitan (>500,000 residents);

*^a^Univariate logistic regression model: no adjustment is necessary or possible – as it would produce a collider bias – to estimate the total effect of the independent variable on the outcome.*

*^b^Multivariable logistic regression model adjusted for the variable: age.*

*^c^Multivariable logistic regression model adjusted for the variables: age, migration background.*
